# The Pan-University Network for Global Health: framework for collaboration and review of global health needs

**DOI:** 10.1186/s12992-016-0151-2

**Published:** 2016-04-21

**Authors:** M. S. Winchester, R. BeLue, T. Oni, U. Wittwer-Backofen, D. Deobagkar, H. Onya, T. A. Samuels, S. A. Matthews, C. Stone, C. Airhihenbuwa

**Affiliations:** Research Associate, Pan-University Network for Global Health, Pennsylvania State University, University Park, PA USA; Health Policy and Administration, Pennsylvania State University, University Park, PA USA; Division of Public Health Medicine, School of Public Health and Family Medicine, University of Cape Town, Cape Town, South Africa; Department of Biological Anthropology, Medical Faculty, University of Freiburg, Bresigau, Germany; Molecular Genetics, Center of Advanced Studies, Department of Zoology, Savitribai Phule Pune University, Pune, India; Health Promotion and Institutional HIV/AIDS Programme, University of Limpopo, Sovenga, South Africa; Faculty of Medical Sciences, University of the West Indies, Cave Hill, Barbados; Departments of Sociology, Anthropology, and Demography, Pennsylvania State University, University Park, PA USA; Department of Biobehavioral Health, Pennsylvania State University, University Park, PA USA

**Keywords:** Global health, Network, Urban health, Collaboration non-communicable disease, Infectious disease

## Abstract

In the current United Nations efforts to plan for post 2015-Millennium Development Goals, global partnership to address non-communicable diseases (NCDs) has become a critical goal to effectively respond to the complex global challenges of which inequity in health remains a persistent challenge. Building capacity in terms of well-equipped local researchers and service providers is a key to bridging the inequity in global health. Launched by Penn State University in 2014, the Pan University Network for Global Health responds to this need by bridging researchers at more than 10 universities across the globe. In this paper we outline our framework for international and interdisciplinary collaboration, as well the rationale for our research areas, including a review of these two themes. After its initial meeting, the network has established two central thematic priorities: 1) urbanization and health and 2) the intersection of infectious diseases and NCDs. The urban population in the global south will nearly double in 25 years (approx. 2 billion today to over 3.5 billion by 2040). Urban population growth will have a direct impact on global health, and this growth will be burdened with uneven development and the persistence of urban spatial inequality, including health disparities. The NCD burden, which includes conditions such as hypertension, stroke, and diabetes, is outstripping infectious disease in countries in the global south that are considered to be disproportionately burdened by infectious diseases. Addressing these two priorities demands an interdisciplinary and multi-institutional model to stimulate innovation and synergy that will influence the overall framing of research questions as well as the integration and coordination of research.

## Background

One of the major challenges in the United Nations 2015 Millennium Development Goals is to address inequalities in health. Building capacity by establishing global partnerships via global research networks is a critical step in identifying and addressing areas of inequality [1]. Launched by Penn State University in 2014, the Pan University Network for Global Health (PUNGH) responds to this need by bringing together researchers from six universities across the globe. In this paper we outline our framework for international and interdisciplinary collaboration, as well the rationale for our research areas, including a review of two central themes: urbanization and health and the intersection of infectious and non-communicable disease (NCDs)

The future success of our network is dependent on true collaboration between partner researchers and institutions. We recognize that the insights, skills, and lessons in a partnership can, and indeed should, occur in a bi-directional manner. As has been addressed in *Globalization and Health*’s ongoing series on innovation in global health systems, reverse and global innovation flow create possibilities for relevant community-based research, open access dialogue, and reciprocity and respect [2–5]. At a fundamental level, this is a challenge to the traditional dynamics of north–south models of collaboration. The global flow of ideas in the realm of health is by no means new, though typically only tacitly acknowledged when flowing in “reverse” [5].

The PUNGH approach addresses the need for multi-level, multi-pronged strategies to improve global health prevention, care, and management [6]. PUNGH research focuses on multiple factors including the health care systems, families, and individual behaviors. The network structure offers opportunities to share progress, results, successes, and challenges, in order to iteratively and constructively propose future global health systems research. The network also makes it possible to assess the utility and generalizability of emerging theoretical frameworks that may be useful in guiding research on urban health and the intersections of global infectious diseases with non-communicable diseases [7].

## Review

### Network background and approach

#### Background

The development of PUNGH stems from efforts to globalize university learning and health systems research. Initiated at Pennsylvania State University through the University Office of Global Programs, it is a thematic Global Engagement Network, designed to build collaborations with key university partners in different parts of the world around the topic of global health. At the inaugural meeting that was held at Penn State in May 2014, 60 faculty members from 13 universities participated in the 2 day deliberations. With the understanding that research and training/education are best addressed via a global collaboration, groups of scholars reviewed a set of global health topics with the goal of identifying those with the greatest interest and opportunities. At the end of the two days, two priorities were established for the network: 1) urbanization and health and 2) the intersection of infectious diseases with NCDs.

The members of PUNGH have committed to addressing deficiencies in global health research and sharing resources. One of the objectives of the network is to link research data from various network members and make resources available to all members. Another is to create innovative research projects and new collaborative teams. The network facilitates activities that addresses current deficiencies in research at all levels, as called for by local populations, as well as training and capacity building. We plan to engage communities in the creation of future research agendas. At our most recent meeting in Cape Town, in October 2015, we began to interface with local government representatives. PUNGH members presented their preliminary findings from network-sponsored projects and dialogues with representatives regarding their current activities and identified needs for health-related research in the region.

Capacity building and advocacy are also key tenets of the network and central to bridging the divide between academic researchers and healthcare practitioners. Capacity building includes teaching, research, training, and innovative educational programs, rolled out over the next 5 years. Through advocacy of global health the network will engage researchers from other fields or those looking to refocus their careers, influence students to study global health, and collect information on global health that would be useful to health policymakers and planners and package that information in a manner that is meaningful and useful to them.

#### Activities

**Fig. 1:**
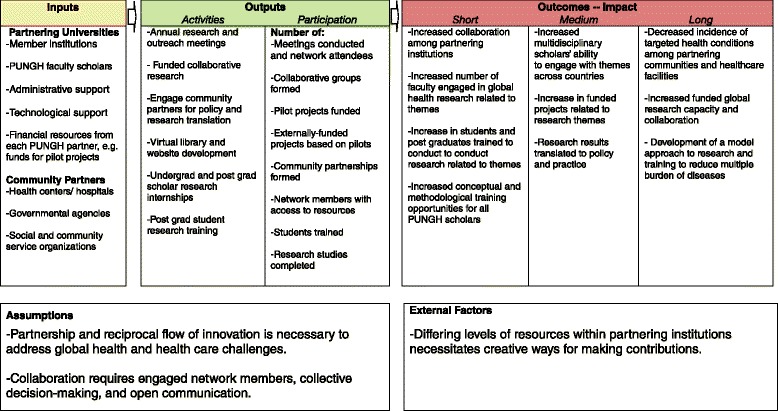
Pan University Network for Global Health logic model

Still early in its formation, the PUNGH is in the process of mapping out activities for the coming years. The network is housed at Penn State University and champions have been identified from each of the six core anchor institutions. These leaders meet on a quarterly basis to update each other on their respective campus activities and upcoming events. An annual meeting of network members is hosted on a rotating basis by the anchor institutions: Penn State University (2014), University of Freiburg (2014), University of Cape Town (2015), Savitribai Phule Pune University (2016), and in future cycles by the University of the West Indies and University of Limpopo. The PUNGH is guided by the following logic model to guide network activities, to use as a basis for network evaluation and program development (see Fig. 1). Although PUNGH membership is currently limited to universities, we view input as coming from institutions, individuals, community members, government agencies, and social and community service organizations. Similarly, outputs reach beyond the number of projects funded to incorporate partnerships, access to resources, and factors such as community participation and student engagement. As a research network, we hope to develop enduring partnerships, as reflected in the outcomes to be assessed over the short, medium, and long term.

Between meetings, researchers share ideas, articles, and other resources through the webpage and online exchanges. We are in the process of creating an online library of sources from network members. Currently, six pilot projects have been funded by the network and are underway to solidify the new collaborations and collect information for future studies. These projects (grouped by theme) include:

Multiple morbidities:

- Intersection of HIV/AIDS and NCDs, focusing on Cardiovascular Diseases: Creating Collaborative Teams in the West Indies and South Africa
- Strengthening Health Systems for Chronic Care: Intersection of Communicable and NCD Services in South Africa
- Obesity Paradox: Body Mass Index and Mortality in US and Asian Older Adults

Urbanization and Health:

- Development of a Multidisciplinary Network of Established and Emerging Scholars on Migration, Urbanization and Health in Southern Africa
- The Impact of Urbanization on Vitamin D Deficiency and Adverse Pregnancy Outcomes in India
- Identifying Urban Transition Priority Areas for Mother and Child Interventions in Cape Town and India

These projects address a range of pressing global health issues and local contexts within our thematic areas and have significant implications for addressing health system deficiencies. Researchers come from multiple disciplines including: public health, health policy, nutrition, biological anthropology, biobehavioral health, medicine, demography, geography, sociology, and environmental science. Each project aims to expand into a larger collaborative initiative among researchers to develop new protocols, train students in research methods, engage the community with research, and develop clinical and policy recommendations from findings. External support for the next phases of research is being sought for each individual project and the funds will be shared among the collaborating institutions. Initial findings were presented and discussed at the most recent network meeting, a process which will be repeated annually.

#### Benefits and challenges

There are compelling advantages to having institutional partnerships so that individuals can collaborate to have a sustainable impact in research and educational activities compared to working individually within institutions, though we do note that there are many potential challenges as well [8]. The following specific advantages have been identified by network members, many of which are reflected in our logic model and group processes (see Fig. 1):

- Drawing on collective expertise to respond adequately to important global needs in research and training.
- Sharing successful and unsuccessful research methods to develop effective globally effective practices.
- Attracting and organizing global health researchers from our partner institutions by promoting a long-term goal based on a culture of collaboration in the network.
- Supporting local workshops that involve community participation in data collection in research that responds to local needs. This should help to overcome the academic dilemma of typically developing research objectives without engagement of the end-user.
- Promoting a holistic approach to research and training particularly when addressing global health issues that are related to complex physical and social stressors.

The creation of a collaborative network is not without challenges and we regularly engage in critical reflection regarding processes, decision-making, and barriers. Two of the biggest challenges to PUNGH so far have been communication and resources. At our most recent meeting, we redesigned the steering committee to be more inclusive and promote open communication between all network partners. While we plan to scale up funding for research projects, resources will continue to be an issue, particularly for partners in Lower and Middle Income Countries (LMICs).

PUNGH members represent a variety of diverse institutions and communities. Conducting multi-national collaborative research requires knowledge of applicable science, but also of how to negotiate various institutional and community cultures. In response, network members have built in time to meet via phone, video conferencing or in-person to discuss these issues and to encourage learning about our various institutional and community histories, policies and cultures. These processes also help to maintain group focus on the two current thematic priorities reviewed below.

### Urbanization and health

The topic of urbanization and health has generated several recent reviews [9–12] and as recently as 2010 was the core theme of the World Health Organization’s World Health Day. The synergy between urbanization and new burdens of disease [13, 14] coupled with the rapid pace of change requires our immediate attention. PUNGH is committed to the study of urbanization and health now because within 30 years our urban world will become an even more complex mosaic of risk/protection [15].

Population estimates suggest that by 2045 there will be over nine billion people on the planet, of which over six billion will be urban residents. The continental, country and within-country variation in the trends we see today relating to shifting demographics and spatial inequalities will ensure that the bulk of the population growth will occur in developing countries, with relative growth being highest across Africa. While megacities (the very largest global metropolises) are often highlighted, urban growth has occurred across the entire settlement system, reinforcing existing health challenges as well as generating new ones [16]. Processes of urbanization provide the dynamic backdrop to how we conceptualize and define global health challenges. The PUNGH network explicitly acknowledges the role of urbanization on human health.

Health and mortality transitions associated with urbanization and related processes have helped redefine the health and disease profiles of nations, states, and local contexts across the globe [17]. Changes in health, fertility, age-structure, migration and urbanization are all interconnected, and influence family and household transitions, which reinforce the need not just to focus on population growth but on the compositional heterogeneity of populations. There is a clear need for researchers and policy makers alike to focus on vulnerable populations defined by age, gender, ethnicity, and socioeconomic status, as well as vulnerable subgroups, and how these groups adapt to changing urban landscapes across the lifespan [18, 19]. More generally, a focus on interconnected demographic and health transitions positions our conceptual framing of urbanization processes within the macrosocial determinants of health perspective [20]. And, a focus on population heterogeneity or subpopulations will facilitate our examination of social gradients in health and well-being [21], urban spatial inequalities [22] and health equity and health disparities [23]. As Weeks et al. [22] have shown in their study of urban slums in Ghana, it is critical that we better understand the processes underlying the dimensions of spatial inequality in a rapidly changing urban environment. Their work shows a heterogeneity of risk for disease and points to a need to disaggregate labels of place, such as ‘slum’ as even within these spaces of risk there are relative winners and losers, or social gradients in health [21].

The magnitude and speed of urban change is particularly evident in the Southern African Development Community (SADC) region, where the population is expected to double to approximately 500 million by 2040 (medium variant, UN Statistical Division) and both internal and cross-border migration will redistribute the region’s population to urban centers. Growing poverty, inequality in resource access, and shifting risk exposures represent important challenges. Addressing such challenges requires new interdisciplinary discussions to improve research and policy responses to health migration in the context of inequality at local, regional, and national scales. One of our SADC-based pilot projects is bringing together an interdisciplinary group of emerging and established scholars to forge collaborative, cross-continental, and multidisciplinary studies on urban health in the context of migration [24, 25]. While the substantive topic of migration is the linchpin bringing together scholars from multiple backgrounds, this group will examine opportunities for methodological and empirical linkages across disciplines that will allow for the development of innovative study designs and data collection efforts as part of competitive grants around migration, urbanization and health in southern Africa. A key innovation in this research group is the involvement of, and commitment to mentoring, emergent African scholars.

While urbanization has been a dominant force shaping human life across many parts of the globe, a new disease landscape also has emerged from persistent trends in health and mortality—the epidemiological transition [26]; this new disease landscape is one in which NCDs are in the ascendancy.

### Intersection of Infectious Diseases (ID) and Non-Communicable Diseases (NCD)

Historically, the concept of the epidemiological transition was used to describe how with economic development death rates from infectious diseases tend to fall, especially in infants and children, fertility declines, the population starts to age, and NCDs become the predominant health problems- over a period of centuries [26]. In today’s globalized world, however, many LMICs are undergoing rapid changes that are associated with continuing high levels of infectious diseases, while concomitantly developing high rates of NCDs [14]. Rapid urbanization, mechanization of the rural economy, and the increasing activities of trans-national food, drink and tobacco corporations are all associated with behavioral changes that increase the risk of NCDs [27]. As a result, population health profiles and patterns are rapidly changing with an increase in cardiovascular and metabolic disorders.

In most of sub-Saharan Africa, the leading risk factors for disease are childhood malnutrition and air pollution; whilst in southern Africa, Eastern Europe and South America, alcohol use is the leading risk factor [27]. The prevalence of overweight/obesity, a risk factor for cardiovascular disease and type II diabetes mellitus (T2DM), has risen by 82 % since 1990. High body mass index (BMI) is now a more important cause of morbidity than childhood malnutrition both globally and in many LMICs [28]. Furthermore high blood pressure and tobacco smoking are ranked in the top five risk factors across most LMIC regions. This epidemiological transition is also observed in LMICs in the causes of disability adjusted life years (DALYs), a measure of disease burden based on combining premature mortality and disability [29]. When considering mortality alone, 90 % of persons dying from NCDs who are under the age of 70 live in LMICs [30]. Globally, NCDs already account for over 65 % of deaths, and 58 % of deaths in developing regions. Sub-Saharan Africa is the only region where deaths from communicable disease still outnumber those from NCDs, and by more than 2 to 1. In addition, the disease burden and deaths are occurring at younger ages than in high-income countries resulting in a loss of economic output [31, 32] and increasing household expenditure associated with management of chronic disease.

A good understanding of the burden of disease and risk factors is important because in addition to these conditions co-existing [33], diseases, disease precursors, and risk factors can also interact influencing host susceptibility, clinical manifestation, and disease prognosis, further impacting on population health. Some NCDs have partially communicable causes, while many communicable diseases are associated with NCD sequelae. Interactions between communicable diseases and NCDs are complex and often mediated by shared risk factors. This highlights the limitation of the broad classification of diseases into discrete categories, a potential barrier to the management of patients with complex comorbid infections, at both the provider and health system level [34]. The change in disease patterns should therefore be accompanied by changing research priorities to effectively improve population health.

The PUNGH is uniquely positioned to study NCD/ID multimorbidities as the partnership engages countries across income levels. This perspective allows us to examine the NCD/ID epidemic at various stages of epidemiologic transition and identify and share strategies used to manage multiple morbidities in multiple settings. Furthermore, we are able to explore several key NCD/ID combinations through various network research projects including the comorbidity of HIV and cardiovascular disease in the West Indies, US and South Africa, HIV and diabetes in rural urban South Africa and the association between obesity and ID in India and China. In addition, we are in the process of documenting the extent that multiple morbidity affect these diverse populations given that NCD/ID multimorbidity often encompass more than two conditions [35, 36].

## Conclusion

Global health challenges are complex and therefore require institutional partnerships for research and training. PUNGH, a relatively smaller global network of institutions, has laid the foundation with a focused priorities on urbanization and ID/NCDs to respond collectively to the growing multiple burden of diseases. PUNGH has invested in a mechanism to incentivize multidisciplinary research collaborations which is a cornerstone to building and strengthening research capacity for global health.

In the future, the network plans to expand membership to additional universities with cadres of global health researchers, though not until we have institutional commitment among the founding members and expanded current research activities. The initial phases of network activity have generated momentum and engagement among members, which we recognize as key to continuing the partnership into the future. Despite challenges in communication and resources, we believe that our commitment to engaging scholars across region, discipline, and career stages will create a long-ranging model of research collaboration. Ultimately, a commitment to mutual respect and value between the global north and south, a core value of PUNGH, is critical if we are to have a sustainable impact on reducing the multiple burdens of diseases globally.

## References

[1] Airhihenbuwa CO, Shisana O, Zungu N, BeLue R, Makofani DM, Shefer T (2011). Research capacity building: a US-South African partnership. Glob Health Promot.

[2] Binagwaho A, Nutt CT, Mutabazi V, Karema C, Nsanzimana S, Gasana M (2013). Shared learning in an interconnected world: innovations to advance global health equity. Global Health.

[3] Crisp N (2014). Mutual learning and reverse innovation--where next?. Global Health.

[4] Snowdon AW, Bassi H, Scarffe AD, Smith AD (2015). Reverse innovation: an opportunity for strengthening health systems. Glob Health.

[5] Syed SB, Dadwal V, Martin G (2013). Reverse innovation in global health systems: towards global innovation flow. Global Health.

[6] Amuyunzu-Nyamongo M (2010). Need for a multi-factorial, multi-sectorial and multi-disciplinary approach to NCD prevention and control in Africa. Glob Health Promot.

[7] Shippee ND, Shah ND, May CR, Mair FS, Montori VM (2012). Cumulative complexity: a functional, patient-centered model of patient complexity can improve research and practice. J Clin Epidemiol.

[8] Barry MM, Allegrante JP, Lamarre MC, Auld ME, Taub A (2009). The Galway Consensus Conference: international collaboration on the development of core competencies for health promotion and health education. Glob Health Promot.

[9] Galea S, Vlahov D (2005). Urban health: evidence, challenges, and directions. Annu Rev Public Health.

[10] Gong P, Liang S, Carlton EJ, Jiang Q, Wu J, Wang L (2012). Urbanisation and health in China. Lancet.

[11] Eckert S, Kohler S (2014). Urbanization and health in developing countries: a systematic review. World Health Popul.

[12] Nonini D (2014). A Companion to Urban Anthropology.

[13] Murray CJ, Lopez AD (1997). Global mortality, disability, and the contribution of risk factors: Global Burden of Disease Study. Lancet.

[14] Murray CJ, Lopez AD (2013). Measuring the global burden of disease. N Engl J Med.

[15] Fitzpatrick K, LaGory M (2000). Unhealthy places: The ecology of risk in the urban landscape.

[16] Montgomery MR (2008). The urban transformation of the developing world. Science.

[17] Weeks J (2012). Population: An introduction to concepts and issues (11th Edition).

[18] Chant S (2013). Cities through a “gender lens”: a golden “urban age” for women in the global South?. Environ Urban.

[19] Harpham T (2009). Urban health in developing countries: what do we know and where do we go?. Health Place.

[20] Galea S (2007). Macrosocial determinants of population health.

[21] Marmot M (2004). The Status Syndrome: How Social Standing Affects Our Health and Longevity.

[22] Weeks J, Hill A, Stoler J (2012). Spatial Inequalities: Health, Poverty, and Place in Accra, Ghana.

[23] Friel S, Akerman M, Hancock T, Kumaresan J, Marmot M, Melin T (2011). Addressing the social and environmental determinants of urban health equity: evidence for action and a research agenda. J Urban Health.

[24] Myroniuk W, Vearey J (2015). Social Capital and Livelihoods in Johannesburg: Differential Advantages and Unexpected Outcomes among Foreign‐Born Migrants, Internal Migrants, and Long‐Term South African Residents. Int Migr Rev.

[25] Vearey J (2014). Healthy migration: a public health and development imperative for south(ern) Africa. S Afr Med J.

[26] Omran AR (2005). The epidemiologic transition: a theory of the epidemiology of population change. 1971. Milbank Q.

[27] Bradshaw D, Dorrington R, Laubscher R. Rapid Mortality Surveillance Report 2011. South Africa: Medical Research Council; 2012. ISBN: 978-1-920618-00-1 Report. http://www.mrc.ac.za/bod/RapidMortality2011.pdf.

[28] Lim SS, Vos T, Flaxman AD, Danaei G, Shibuya K, Adair-Rohani H (2012). A comparative risk assessment of burden of disease and injury attributable to 67 risk factors and risk factor clusters in 21 regions, 1990–2010: a systematic analysis for the Global Burden of Disease Study 2010. Lancet.

[29] Murray CJ, Vos T, Lozano R, Naghavi M, Flaxman AD, Michaud C (2012). Disability-adjusted life years (DALYs) for 291 diseases and injuries in 21 regions, 1990–2010: a systematic analysis for the Global Burden of Disease Study 2010. Lancet.

[30] WHO (2011). Global status report on non-communicable diseases.

[31] Boutayeb A (2006). The double burden of communicable and non-communicable diseases in developing countries. Trans R Soc Trop Med Hyg.

[32] Oni T, Stoever K, Wilkinson RJ (2013). Tuberculosis, HIV, and type 2 diabetes mellitus: a neglected priority. Lancet Respir Med.

[33] Oni T, Youngblood E, Boulle A, McGrath N, Wilkinson RJ, Levitt NS (2015). Patterns of HIV, TB, and non-communicable disease multi-morbidity in peri-urban South Africa- a cross sectional study. BMC Infect Dis.

[34] Oni T, McGrath N, BeLue R, Roderick P, Colagiuri S, May CR (2014). Chronic diseases and multi-morbidity--a conceptual modification to the WHO ICCC model for countries in health transition. BMC Public Health.

[35] Abas M, Ali GC, Nakimuli-Mpungu E, Chibanda D (2014). Depression in people living with HIV in sub-Saharan Africa: time to act. Trop Med Int Health.

[36] Mendenhall E. Beyond Comorbidity: A Critical Perspective of Syndemic Depression and Diabetes in Cross-cultural Contexts. Med Anthropol Q. 2015. doi:10.1111/maq.12215. http://onlinelibrary.wiley.com/doi/10.1111/maq.12215/full. 10.1111/maq.12215 PMC4600415 25865829

